# Association of Healthy Diet and Physical Activity With Breast Cancer: Lifestyle Interventions and Oncology Education

**DOI:** 10.3389/fpubh.2022.797794

**Published:** 2022-03-23

**Authors:** Tiantian Jia, Yufeng Liu, Yuanyuan Fan, Lintao Wang, Enshe Jiang

**Affiliations:** ^1^Institute of Nursing and Health, Henan University, Kaifeng, China; ^2^School of Life Sciences, Henan University, Kaifeng, China; ^3^Department of Neurology, The First Affiliated Hospital of Henan University, Kaifeng, China

**Keywords:** breast cancer, diet, physical activity, oncology education, lifestyle

## Abstract

Global cancer statistics suggest that breast cancer (BC) is the most diagnosed cancer in women, with an estimated 2. 3 million new cases reported in 2020. Observational evidence shows a clear link between prevention and development of invasive BC and lifestyle-based interventions such as a healthy diet and physical activity. The recent findings reveal that even minimal amounts of daily exercise and a healthy diet reduced the risk of BC, mitigated the side effects of cancer treatment, and stopped the recurrence of cancer in the survivors. Despite the myriad benefits, the implementation of these lifestyle interventions in at-risk and survivor populations has been limited to date. Given the need to disseminate information about the role of physical activity and nutrition in BC reduction, the review aimed to present the recent scientific outreach and update on associations between the lifestyle interventions and BC outcomes to narrow the gap and strengthen the understanding more clearly. This review covers more direct, detailed, and updated scientific literature to respond to frequently asked questions related to the daily lifestyle-based interventions and their impact on BC risk and survivors. This review also highlights the importance of the oncology provider's job and how oncology education can reduce the BC burden.

## Introduction

Breast cancer (BC) is a growing global public health concern. Although many high-income countries have reported decreases in BC mortality due to the advances in treatment, diagnosis, and implantation mammographic screening (1), BC mortality is steadily increasing in most developing countries due to low health knowledge and limited medical facilities (2–4). According to the recent reports, ~2.3 million new cases of BC are diagnosed each year, with a mortality rate of roughly 4,50,000 per year (5, 6). The leading risk factors for BC include age, genetic mutations (BRCA1 and BRCA2) (7), lifestyle-based (non-genetic) risk factors (8), early menarche, nulliparity, first pregnancy after the age of 30 years, older age at menopause, dense breast tissue (9), hormone replacement therapy (10), use of oral contraceptives (11), personal and family history of BC, and other clinical complaints (12, 13).

In cancer education, it is challenging but fundamentally important to accurately evaluate the role of modifiable risk factors, such as diet, physical activity, and other non-genetic factors, to estimate BC risk for individual women—the first essential step toward precision prevention.

Evaluating the complex interaction between diet and physical activity and better BC outcomes resulted in a significant reduction in BC incidence and enhanced survival rates (14, 15). Substantial evidence underlines the importance of a balanced diet and increased physical activity to prevent BC and reduce the chances of recurrence and mortality in both pre- and post-menopausal women (16, 17). Currently, exercise and nutrition are considered as two integrative therapy components that play a crucial role in relieving the side effects of active cancer treatment and especially cancer-related fatigue (18). The World Cancer Research Fund recommends specific guidelines of moderate aerobic exercise for muscle strengthening and nutrition, that is, eating more plant-based foods, limiting red and processed meat, limiting energy-dense foods, salt, sugary drinks, and alcohol, and not relying on dietary supplements (19). The published literature has elaborated on the role of physical activity in improving fitness, reducing psychological distress, and enhancing cognitive abilities (20–24), whereas nutritional consultations may help manage challenges associated with anemia, diarrhea, nausea, and vomiting which contribute to cancer-related fatigue (25–27).

Strategies are needed for disseminating literature on the association between BC and physical activity and nutrition. Thus, this review summarizes and reviews the evidence linking lifestyle factors—especially diet and exercise—to BC risks and outcomes. The purpose of this narrative review is to present recent scientific outreach and update on associations between the lifestyle interventions and BC outcomes to narrow the gap and strengthen the understandings more clearly. In comparison with the published studies on the subject, the current review covers more direct, detailed, and updated scientific literature to respond to frequently asked questions related to the daily life style-based interventions and their impact on BC risk and survivors. This review also highlights the importance of the oncology provider job and how oncology education can reduce the BC burden.

## Search Strategy and Selection Criteria

We performed a literature search using the different search databases, PubMed, Google, Google Scholar, and Research Gate with the key words of BC, physical activity, exercise, diet, food menu, and other related terms. In addition to the relevancy of the title and abstracts, for strong conceptual and logical understanding, more recent studies were selected for inclusion based on the year of publication between 2016 and 2021. Though minor older publications and some news agencies, government data reports were also cited to strengthen the subject's background. All selected articles have been cited accordingly.

## Dietary Factors in BC Incidence and Recurrence

Adhering to a healthy lifestyle in terms of high-quality food intake influences the risk of BC onset and post-diagnosis outcomes. Epidemiological and preclinical data suggest that some food and nutrients (e.g., saturated fats, red and processed meat) increase the circulating levels of endogenous estrogens, insulin-like growth factors, and pro-inflammatory cytokines, thus supporting BC development. In contrast, polyunsaturated fatty acids, vitamins C and E, fresh fruits, and vegetables have protective effects against BC onset or progression (28, 29). Several studies report an inverse relationship between BC and the Mediterranean diet. However, there is insufficient information to date to confirm the interplay of diet and BC incidence and mortality (30–33).

### Fruits and Vegetables

**Figure 1:**
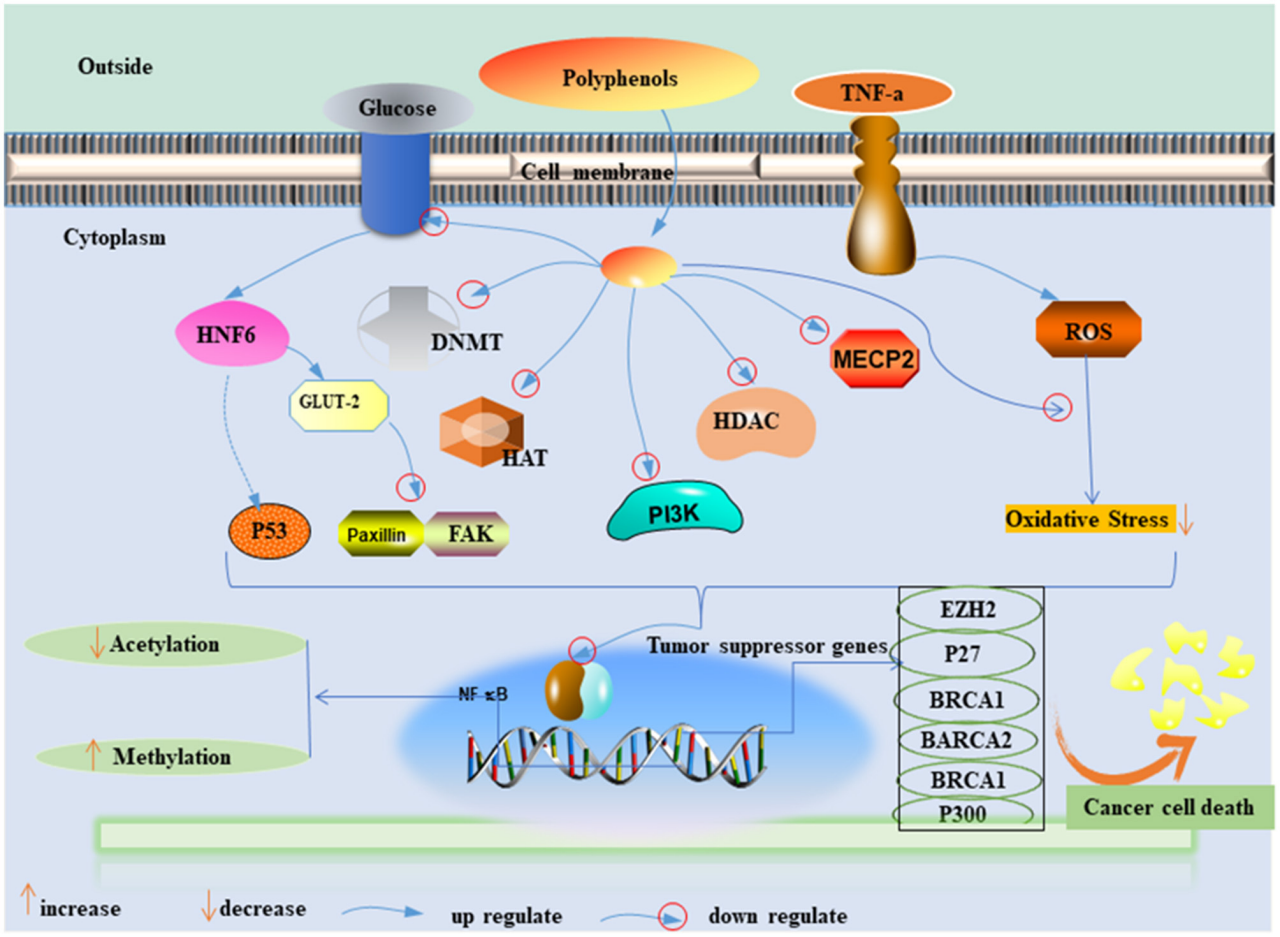
Possible mechanisms of polyphenol effect on the inhibition of BC cell proliferation. PI3K, phosphoinositide-3–kinase; TNF a, tumor necrosis alpha; ROS, radioactive oxide species; NF-κB, nuclear factor kappa light chain enhancer of activated B cells; DNMT, DNA methyltransferase; HAT, histone acetyltransferases; HMT, histone methyltransferases; HDAC, histone deacetylases; GLUT-2, glucose transporter-2; MeCP, methyl CpG binding protein; EZH2, enhancer of zeste homolog 2; BRCA1, 2, breast cancer 1, 2; HNF- 6, hepatocyte nuclear factor 6; FAK and paxillin, two focal adhesion–associated proteins.

Fresh fruits and vegetables are known as natural antioxidants. Their routine consumption increases the consumer polyphenols and fiber level in the body, which provides resistance against tumorigenesis (34–36). Polyphenols have the potential to modulate the proliferation and metastatic activity of BC by regulating the cellular signaling pathways and inhibiting the enzymatic activity of tumor-supportive proteins such as the transcriptional factor NF-B (Figure 1) (37–39). Polyphenols can also antagonize the estrogen-signaling pathway by inhibiting aromatase and the production of estrogen or blocking the estrogen receptors (ERs) (40–42). A similar mechanism has been observed in fibers to prevent carcinogenesis by binding estrogens and reducing their serum levels or by improving insulin sensitivity and reducing weight gain (43, 44). Furthermore, the European Prospective Investigation into Cancer and Nutrition (Italian) study showed an inverse association between high consumption of fruits and leafy vegetables, which include raw tomatoes, and BC risk (45). However, available evidence suggests that further exploration is needed.

### Red Meat

When red meat is cooked at high temperatures, carcinogen compounds such as heterocyclic amines, N-nitroso compounds, and polycyclic aromatic hydrocarbons are released, which potentially mediates the onset of BC (46–48). These compounds, especially heterocyclic amines, are involved in breast tissue-specific estrogen-based carcinogenic activity and support BC progression (49, 50). In other studies, researchers proposed that consuming processed red meat increases free radicals that are further involved in inducing lipid peroxidation (51, 52). Consumption of unprocessed red meat is thought to carry a 6% higher BC risk than not consuming such foods, whereas processed meat consumption is associated with a 9% higher BC risk (53, 54) due to higher levels of heme iron. The complete avoidance of meat is not usually advocated because it is a source of nutrients, such as proteins, iron, zinc, and vitamin B12. The evidence of its role in BC is inconclusive. However, suggestions to limit consumption of processed red meat are almost undoubtedly well-founded.

### Dietary Fat

Menopausal status affects the influence of dietary fat consumption. Dietary fat poses a higher risk of BC in post-menopausal women, whereas for pre-menopausal women, dietary fats appear to have a protective effect (55). A recent study indicated that a high saturated-fat diet increases the risk of BC and, most conspicuously, of receptor-positive cancer, particularly ER+ (56). Cancer science suggests that dietary fats can influence the process of carcinogenesis by modulating intracellular signaling cascades (57). Although a high-fat diet, total cholesterol, and triglyceride levels have mostly been associated with increased risk, the evidence is limited.

### Carbohydrate

Associations between BC and carbohydrates have been evaluated using the quality of the glycemic index (GI) and glycemic load (GL). The results are contradictory and inconclusive (58–61). A metadata study indicates that higher GI is associated with increased BC risk in post-menopausal women, but no effect was found in pre-menopausal women (62). Similarly, positive associations between GL and ER base and BC risk in post-menopausal women were explored in another study. The findings suggested that GL might increase the serum insulin that further mediates BC onset (63, 64). Whereas, the association between carbohydrate intake, GI, or GL and overall BC risk requires further research, glycemic control is advisable.

### Red Wine Drinking

Red wine drinking is a prominent and modifiable lifestyle risk factor for BC. Some studies provide robust evidence that alcohol intake, regardless of the type of beverage (beer, wine, or spirits), and menopausal status are consistently associated with increased BC risk. A dose–response meta-analysis showed that 10 g of ethanol consumption in a single day contributed to a 5% increase in BC risk for pre-menopausal women and a 9% elevated risk for post-menopausal women (65). The findings reveal that ethanol's association with BC development is due to its ability to promote epithelial–mesenchymal transition, tumor growth, and metastasis formation (66, 67). Ethanol consumption causes an increase in estrogen concentration *via* different physiological mechanisms, for example, increased aromatase activity, prevention of estrogen breakdown, reduction of melatonin secretion, and increased hepatic oxidative stress that allows estrogen to exert its carcinogenic effect on breast tissue (68).

### Soy

**Figure 2:**
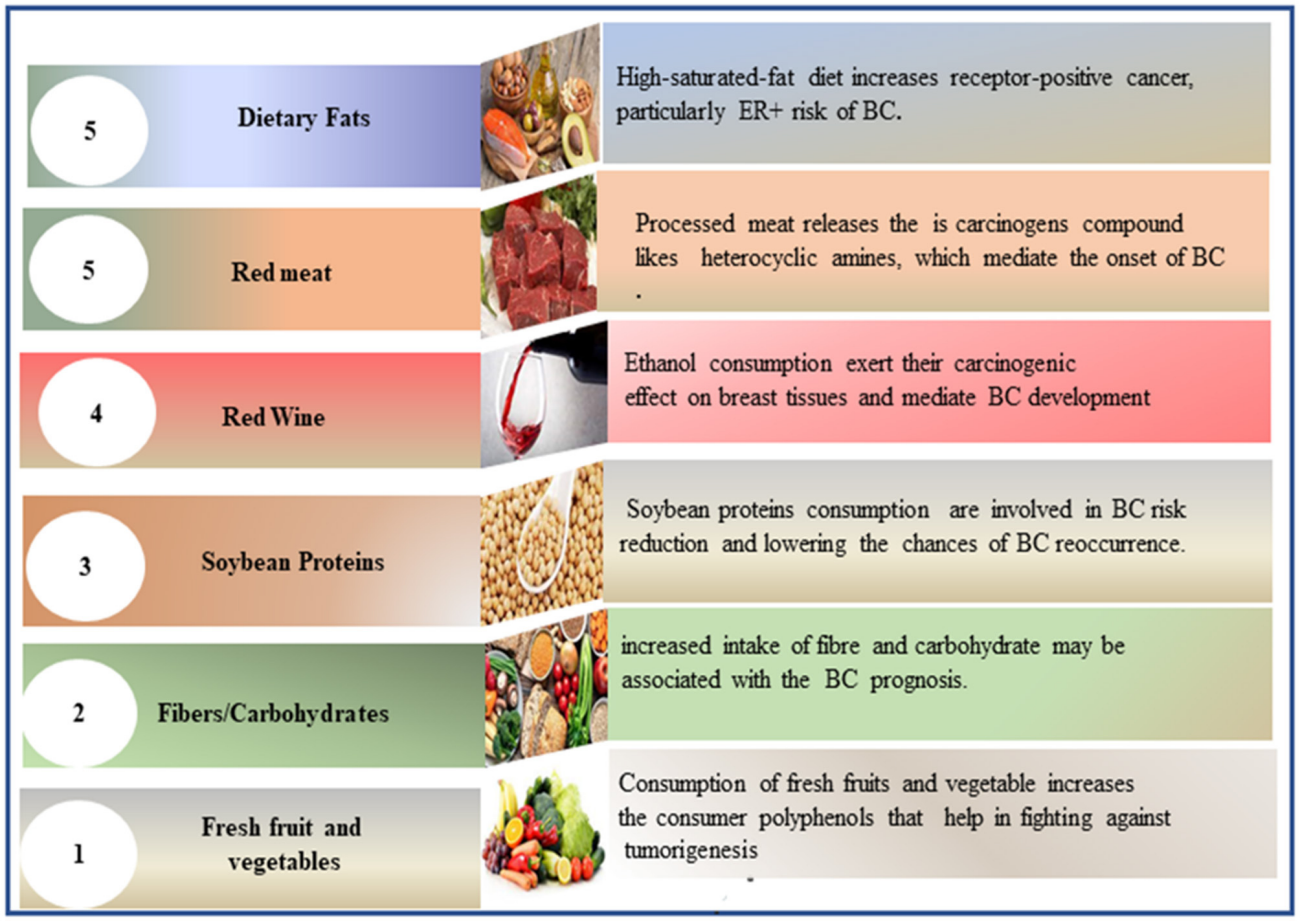
Figurative description of dietary factors involved in BC incidence prevention and recurrence. BC, breast cancer.

Soy is considered an optimal alternative to high-calorie protein sources such as meat. It contains soy isoflavones (known as phytoestrogen) and soy proteins (69). Research reveals that high levels of isolated soy proteins increase insulin-like growth factor 1, which may contribute to BC recurrence (70). The clinical findings indicate that soy protein isolate supplementation slightly stimulates epithelial BC in pre-menopausal women (69, 71). Prospective cohort studies that investigate the role of soy in BC risk reduction and its influence on BC recurrence and mortality rates yielded different results, often in support of soybean protein consumption (72–74). Thus, the relationship between soy consumption and BC remains unclear. Figure 2 provides a figurative description of dietary factors in BC incidence prevention and recurrence.

### Physical Activity and BC

Physical activity has been considered a protective tool against BC since 1980, and the past two decades have witnessed many studies addressing the connection between physical activity and BC (75–77). Several studies have depicted the association between daily physical activity and better health outcomes which further reduce the risk of lifestyle-based risk factors for cancer (75, 78–81). The findings have revealed that physical exercise also lowers the chances of recurrence and is associated with a longer life expectancy in survivors (75, 82, 83). Although there is no direct link between physical activity and cancer risk reduction, however, the multiple interconnected physiological processes, which include sex hormones, insulin resistance and insulin levels, inflammation, oxidative stress, and adipokines, are believed to be involved in lowering the risk of tumorigenesis (84–86). Identifying the processes by which physical activity affects BC risk demonstrates biological plausibility for the observed link and provides evidence for the optimal exercise prescription for cancer risk reduction (15). Furthermore, understanding the exercise and BC link may provide fresh insights into cancer biology, which aids in developing additional preventive and treatment strategies for cancer (86). A more precise understanding of the nature of the association by type, dose, and timing of activity is needed to formulate public health recommendations regarding the association between physical activity and BC risk.

## Physical Activity and BC Prevention

Researchers have well-explored the essential role of exercise in BC prevention and recurrence (87, 88). According to the World Cancer Research Fund/American Institute Cancer Research project findings, higher levels of physical exercise, with a dose–response connection, reduced the incidence of post-menopausal BC (89). In 2020, Physical Activity Guidelines for Americans from the American Cancer Society also advocated leads a physically active lifestyle to prevent and control BC (90). Physical activity influences cancer risk in several ways, which includes metabolic (calorie balance), hormonal, and immunological responses (91–93). Moore et al. analyzed data from the prospective US and European cohorts with self-reported physical activity measurements to evaluate the link between physical activity and the incidence of 26 different cancers. They concluded that physical activity levels are linked to a decreased risk of 13 malignancies, including BC (94). However, the current understanding of important levels of physical activity and their inverse relationships in patients with ER-negative BC needs further exploration. To address the challenges of women's lifestyle interventions and encourage them to be more physically active to lower their BC risk, many healthcare professionals employ technology-based interventions to raise daily physical activity levels (95–97).

## Physical Activity and BC Recurrence and Survival

Regardless of menopausal status, physical activity plays a significant role in reducing the onset of BC and supports survivors to overcome the disease. However, exercise has a more beneficial impact on pre-menopausal women (98, 99). Similar results were reported in a large-scale dose–response relationship study. Findings confirmed the association between physical activity and lowered risk of BC recurrence and increased survival rates. Furthermore, women with ER-positive tumors benefit the most from exercise (93, 100). However, the clinical oncologists can design the particular exercise program and prescription for women. There are three distinct study phases with different exercise prescriptions, which include treatment phase (three times per week for the length of chemotherapy, plus radiation, if received), post-treatment phase (two times per week for the following 10 week), and maintenance phase (one time per week for an additional 10 week) (101). Similarly, in a recent study, it has been found that a supervised therapeutic exercise program plus patient therapeutic education can significantly reduce the perceived fatigue and increase functional capacity in BC survivors suffering from cancer-related fatigue compared to an unsupervised physical exercise program based on individual preferences with patient therapeutic education (102). Taking all together, these findings suggest that physical exercise is a simple, cost-effective way of mitigating BC risk that could affect the outcome of millions of women with BC.

## Breast Cancer Treatments

**Figure 3:**
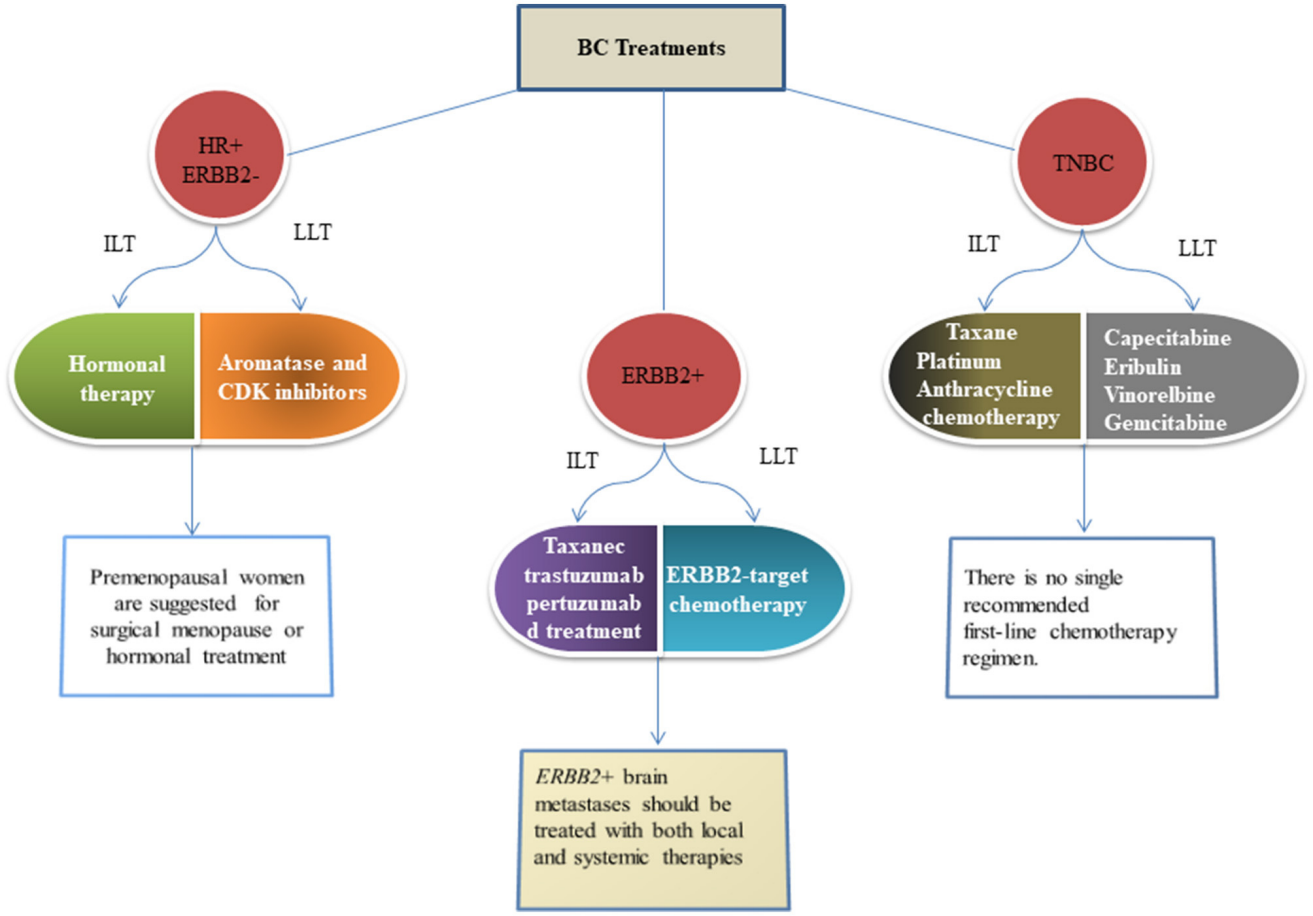
Figures give the recommended standard approaches to therapy of metastatic BC as per the guideline of the American Cancer Society. These recommendations are adopted in (108) and are given as per the guidelines of the National Comprehensives Cancer Network (109). HR, Hormonal receptor; ERBB2, Epidermal growth factor-2; TNBC, Triple negative breast cancer; ILT, Initial line of therapy; LLT, Later line of therapy.

Based on the presence and absence of molecular markers on the plasma membrane of breast cells, BC is categorized into three major subtypes (i) estrogen and progesterone receptor-negative BC (HR-), (ii) human epidermal growth factor 2 (ERBB2) receptor BC, and (iii) both hormonal and growth factor negative (TNBC) (103). Totally, 90% of BC is not metastatic at diagnosis (104). All patients with non-metastatic BC are recommended to undergo local therapy that consists of surgical resection and consider postoperative radiation if lumpectomy is performed. However, patients are advised to undergo systemic therapy before surgery (105, 106). All metastatic BCs are treated according to their subtype with the ultimate goal of prolonging life and palliating the symptoms. Treatments of the 3 BC subtypes have distinct risk profile strategies. Optimal therapy for each patient depends on tumor subtype, anatomic cancer stage, and patient preferences (107). Figure 3 describes the different treatments according to the type of BC.

## Which Lifestyle Modifications Should BE Encouraged?

Analysis of critical findings indicates that lifestyle factors, especially diet and physical activity, have the most robust effect on BC outcomes. A trial-based study showed that adapting routines to include 75 min of vigorous exercise per week and 2–3 weekly strength-training sessions can help to reduce the risk of BC recurrence and mortality (110, 111). To modify lifestyle-related health, these studies appeal to all healthcare professionals to promote and encourage exercise in BC survivors, as receiving advice from an oncologist to exercise will increase patients' physical activity levels (112). Considering the impact of primary care in BC risk reduction and prevention, oncologists should recommend and endorse specific exercise programs and non-pharmaceutical treatments.

**Figure 4:**
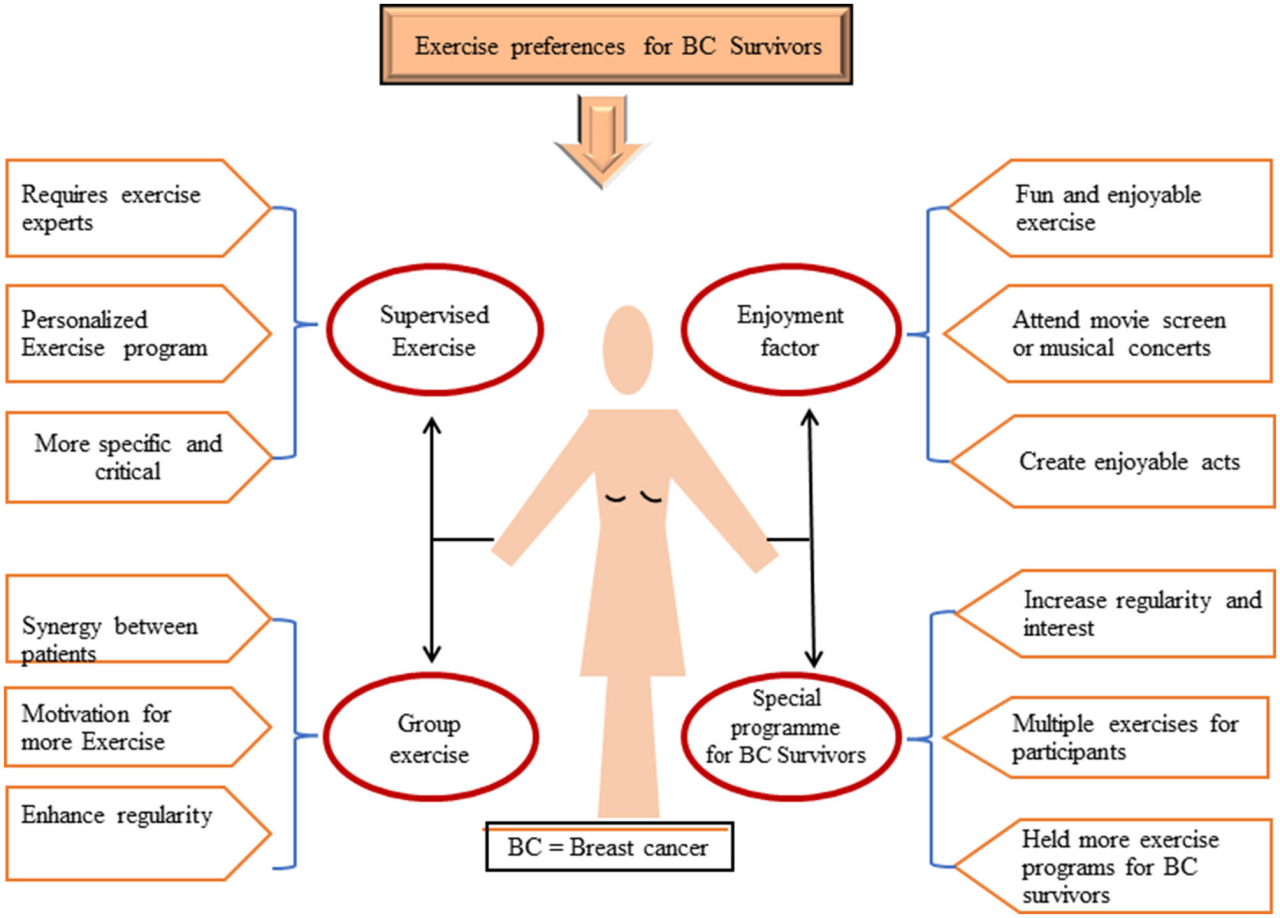
Available preferences of exercises for BC survivors in the form of (i) supervised exercise, (ii) group exercise, (iii) special program for BC survivors, (iv) enjoyment factors. BC, breast cancer.

Clear referral pathways need to be established, so that patients can be directly referred from a cancer care clinic to community-based exercise programs specialized for cancer survivors. Such community-based exercise programs can change individuals' daily routines and result in significant behavioral changes. These interventions can be supported using electronic gadgets or musical instruments for indoor activity, and self-motivation and enjoyment can also be increased through reminders or tracking activities on phones or email (Figure 4) (113). Women who participated in an aerobic exercise program while receiving adjuvant chemotherapy did not gain weight on average, whereas those who did not exercise gained 3.2 kg (114). In addition, exercise advantages include significant self-perceived improvements in attractiveness, cardiorespiratory fitness, lymphedema, and mental wellbeing (115).

While being overweight or obese at the time of diagnosis has a detrimental impact on BC prognosis, it is uncertain if losing weight improves the prognosis of overweight and obese women. To enhance overall survival—and perhaps BC-specific survival—patients should be strongly encouraged to quit smoking (116). Along with physical activity, there is a need to encourage an anticancer diet to help survivors reduce risks. Excessive consumption of western-style diets (high in processed grains, processed meats, and red meats) should be avoided as they are associated with increased rates of BC recurrence (117, 118). There is an association between BC and saturated fat, especially from high-fat dairy products, which may increase BC mortality (119–121). Soy products have not been found to increase BC recurrence and may even reduce it (122, 123).

## Implementing Physical Activity and Diet Management Interventions in Survivorship Care

Considering the clinical importance of developing fundamental guidelines for nutrition and physical activity toward health outcomes, especially BC onset, several international health organizations worldwide have developed the basic guidelines for both general and specific cancer prevention (Box 1) by adopting certain lifestyle interventions. The guidelines for risk reduction and survivorship by The National Comprehensive Cancer Network and The American Cancer Society are widely used by health institutes (124–127). However, incorporating these guidelines into survivorship care is challenging, and the transformation of scientific knowledge into practice in healthcare units is often problematic (128). There are several barriers to disseminate the oncological risk information and its essential association with nutrition and physical activity. These barriers include a lack of knowledge and interest from the general public, scarcity of public education seminars and conferences, uncertainty about when in the cancer continuum to introduce such information, a lack of support from hospital administration, and issues of time and patient flow (129, 130).

**BOX 1:**
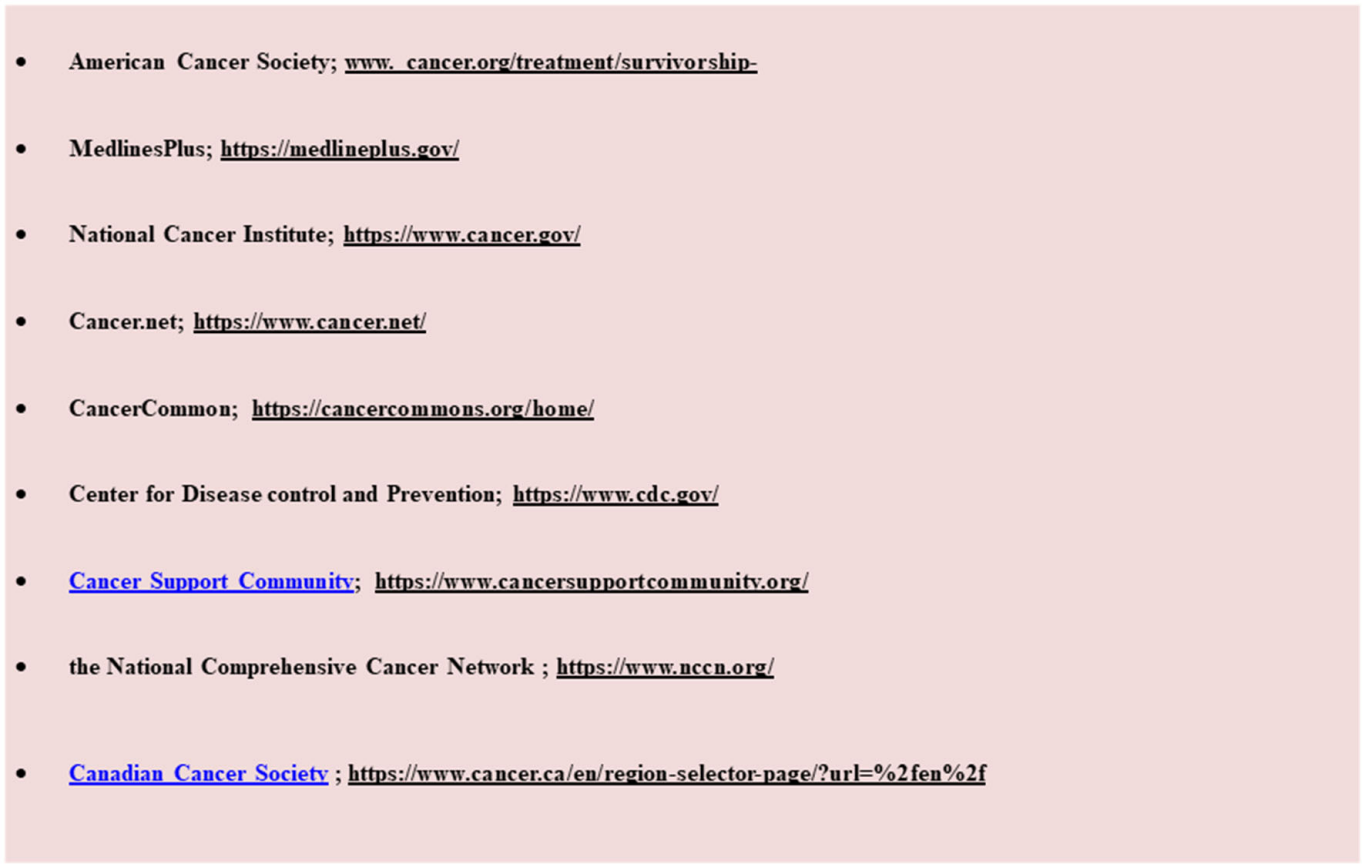
For practices that do not have an in-house patient education department, according to the medical library association, there are several cancer patients' educational websites that provide in-house cancer guidelines for both patients and families.

## Oncology Education

Cancer education suggests that, at a minimum, oncology practice should provide evidence-based information on the association of BC and diet and physical activity in daily life (108). An oncology counselor's services should include answering questions from survivors about whether it is proper for them to be physically active and continue eating specific foods. An oncology counselor needs to address the lack of health education and manage the flow of misinformation from unqualified sources on the internet or within the community (109). They should provide credibly and trusted educational materials that help survivors make informed choices about physical activity and diet (131, 132). Provision of knowledge should be explicit, easily readable, attractive, and available in suitable languages. Survivors need basic information about health behavior recommendations, and patients with BC who cannot access these resources from care units should receive help from in-house patient education by following the advice of web resources mentioned in Box 1.

## Role of Oncology Provider

Risk assessment and counseling of patients on the prevention of BC is highly dependent on the oncology provider. Patients with cancer strongly prefer to receive information about physical activity, diet, and weight management from their oncology providers (133). Studies indicate that oncology providers play a pivotal role and their sessions with patients are very influential (50, 134, 135), which leads to increased physical activity among cancer survivors (136, 137). However, oncology providers may have limited time available for extensive counseling about physical activity, nutrition, and weight management—a further challenge that could be addressed if oncologists themselves advocate around the topic and show the importance of these discussions with their patients. Oncology providers should also become more involved in establishing an advanced infrastructure to reduce BC by emphasizing exercise and diet to cancer survivors and the general public (138). During oncology visits, many care providers demonstrate limited knowledge regarding exercise and diet despite established guidelines from health institutes (139). Considering physical activity and diet interventions as critical components of cancer care, many researchers believe non-pharmacological approaches that the oncology provider's role should include discussion of all and special attention to diet and exercise during patients' chemo-sessions to help support healthy lifestyle changes (140, 141).

To reassure patients who are uncertain about the safety or appropriateness of their diet and exercise regime or weight loss after treatment, comprehensive guidelines should be requested from providers. Furthermore, providers should indicate necessary precautions or additional testing that might be warranted (110). The oncology provider is the healthcare professional well-positioned to show the importance of advocating for cancer interventions, which include lifestyle-related secondary measurements, to cope with the burden of cancer, especially BC.

## Conclusion Remarks

Increasing size of evidence has supported the interplay of BC development with diet and physical activity. Excessive consumption of saturated fats, red meat and red wine, and low intake of fresh fruits and vegetables leads to the accumulation of triglycerides, heterocyclic amines, and ethanol and a lack of polyphenols and fiber levels in the body which mediate the onset of BC and cause an increase in overall mortality in both pre- and post-menopausal women. Similarly, no or minimal physical activity leads to an alteration in sex and metabolic hormones, which raised oxidative stress and inflammation in the body, affecting the menopausal status of women and potentially facilitating the onset of BC. Considering the importance of these interventions, oncology education and the role of oncology providers can play a potential role in developing healthy interventions and reducing the BC burden effectively. Based on these findings, the current review emphasized the value of physical activity and diet management efforts in daily lifestyle to reduce BC risk and improve outcomes.

## Author Contributions

All authors made substantial contributions to conception and design, took part in drafting the article or revising it critically, agreed to be accountable for all aspects of the work, read and edited the submitted version of the manuscript, and approved to submit it to the current journal.

## Funding

This study was supported by the National Natural Science Foundation of China (no. 81900375) and Henan Province Key R&D and Promotion Special (Scientific and Technological Research) Project (no. 212102310147).

## Conflict of Interest

The authors declare that the research was conducted in the absence of any commercial or financial relationships that could be construed as a potential conflict of interest.

## Publisher's Note

All claims expressed in this article are solely those of the authors and do not necessarily represent those of their affiliated organizations, or those of the publisher, the editors and the reviewers. Any product that may be evaluated in this article, or claim that may be made by its manufacturer, is not guaranteed or endorsed by the publisher.
